# Supplementary material for: Restoring Genetic Resource through In Vitro Culturing Testicular Cells from the Cryo-Preserved Tissue of the American Shad (Alosa sapidissima)
Source: Biology (Basel). 2022 May 22;11(5):790. doi: 10.3390/biology11050790 (PMC9139001; doi:10.3390/biology11050790)
Supplement: Supplementary file 1 [file biology-11-00790-s001.zip › biology-1694825-supplementary.pdf]

a.

Age (month)	Body weight (g)	Average gonad index (%)
**7**	250.0-300.0	0.58
**15**	550.0-625.0	2.08
**24**	628.0-850.0	6.46

b.

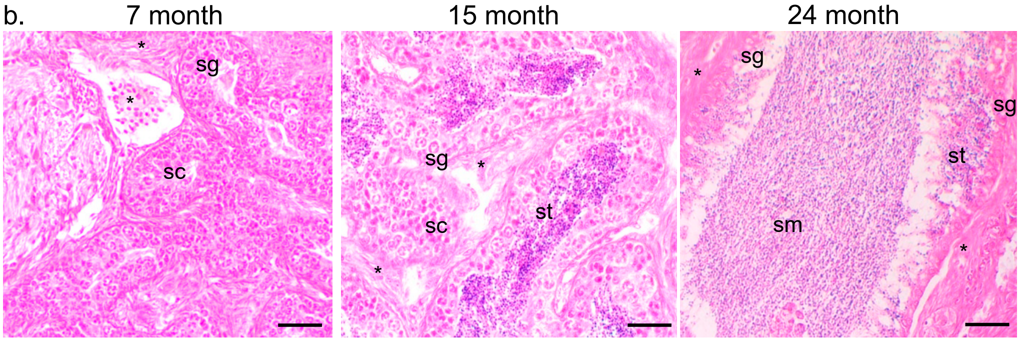

Figure S1. Development of testis in American shad

a. Totally, 30 fish including 10 fish at each age were determined for the gonad index of testis at different age. b. the cellular composition of testis at different ages. sg, spermatogonia. sc, spermatocytes. st, spermatids. sm, spermatozoa and somatic_cells_indicated_with_asterisks. Scale bars, 50 μm.

Table S1. The cell composition of post-thaw American shad testes

Cell types	Proportions of cells (%)			
	7-month-old		24-month-old	
	Isolated cells	Tissue blocks	Isolated cells	Tissue blocks
Spermatogonia	26.0 ± 6.0	28.9 ± 0.2	13.2 ± 0.9	16.1 ± 1.2
Spermatocytes	47.8 ± 5.8	39.4 ± 3.9	7.6 ± 0.5	10.5 ± 1.0
Spermatids and other cells	19.2 ± 0.2	31.8 ± 3.7	79.2 ± 0.4	73.4 ± 2.6

The testicular cells were dissociated fresh testis of 7-month-old and 24-month-old, stained by Hoechst 33342 (MP Biomedicals), PI/Vasa for a brief determination of cell composition testicular cells， and the cells were briefly classified into 3 groups, including spermatogonia, spermatocytes, spermatids and other cells. The data are shown in percentage (%) and the mean ± SEM, n=12 (3 fish x 2 tubes of samples x 2 counts).

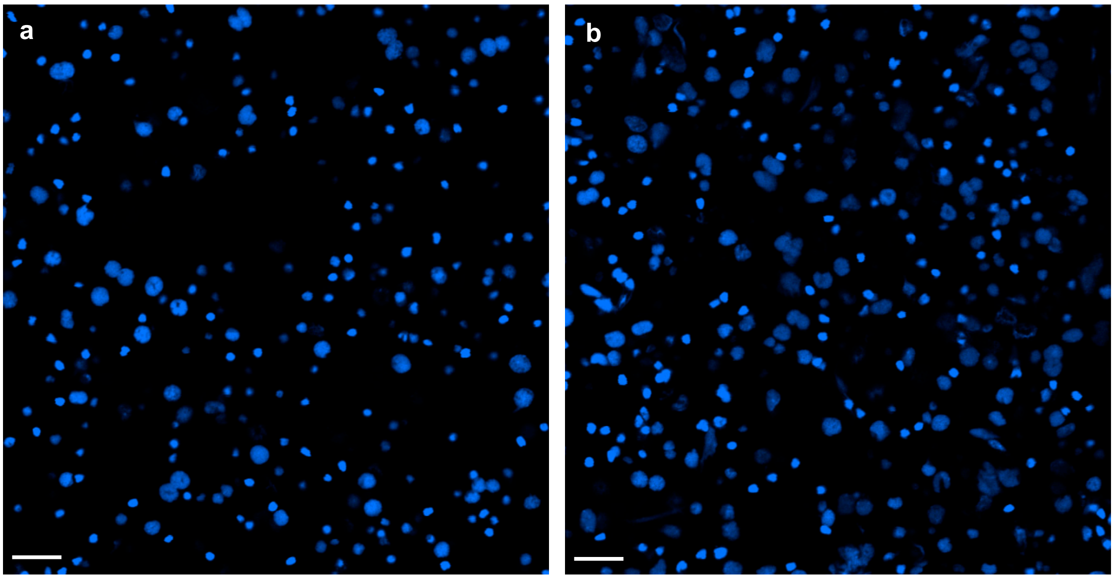

Figure S2. The cell dissociation of cryopreserved cells and testis tissues in American shad

For a pre-check of cell dissociation, the testicular cells were stained by Hoechst 33342（MP Biomedicals) after cryopreservation (a), and isolated from the frozen testis tissues, then stained by Hoechst 33342 (b). Scale bars, 20 µm.

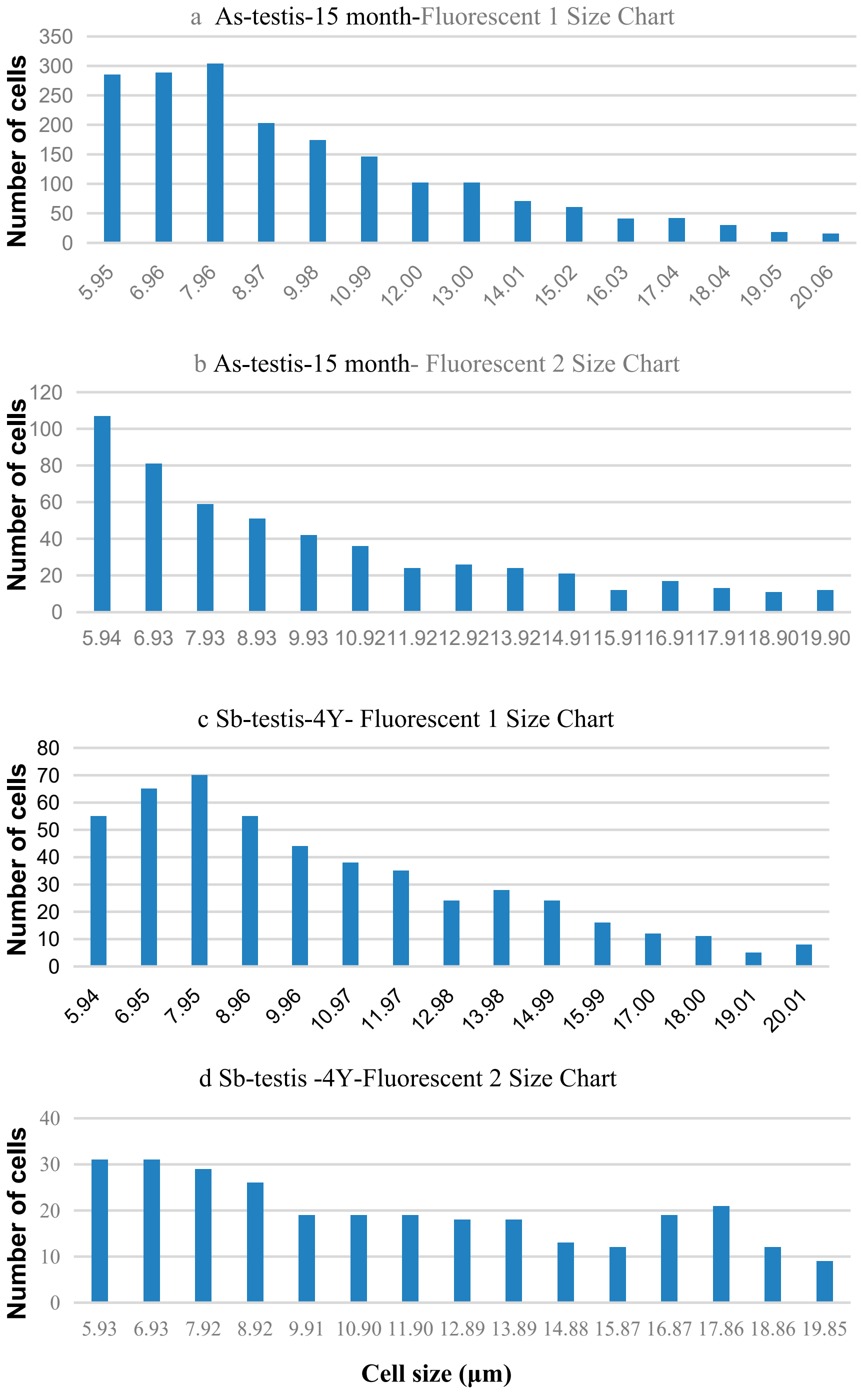

Figure S3. The number of cells at different sizes counted by the Cellometer
